# 11β-HSD1 inhibition ameliorates diabetes-induced cardiomyocyte hypertrophy and cardiac fibrosis through modulation of EGFR activity

**DOI:** 10.18632/oncotarget.22015

**Published:** 2017-10-24

**Authors:** Chunpeng Zou, Weixin Li, Yong Pan, Zia A. Khan, Jieli Li, Xixi Wu, Yi Wang, Liancheng Deng, Guang Liang, Yunjie Zhao

**Affiliations:** ^1^ Chemical Biology Research Center, School of Pharmaceutical Science, Wenzhou Medical University, Wenzhou, Zhejiang, China; ^2^ Department of Ultrasonography, The Second Affiliated Hospital, Wenzhou Medical University, Wenzhou, Zhejiang, China; ^3^ Affiliated Yueqing Hospital, Wenzhou Medical University, Wenzhou, Zhejiang, China; ^4^ Department of Pathology and Laboratory Medicine, Western University, London, Ontario, Canada

**Keywords:** diabetic cardiomyopathy, 11beta-HSD, glucocorticoids, fibrosis, EGFR

## Abstract

11β-HSD1 has been recognized as a potential therapeutic target for type 2 diabetes. Recent studies have shown that hyperglycemia leads to activation of 11β-HSD1, increasing the intracellular glucocorticoid levels. Excess glucocorticoids may lead to the clinical manifestations of cardiac injury. Therefore, the aim of this study is to investigate whether 11β-HSD1 activation contributes to the development of diabetic cardiomyopathy. To investigate the role of 11β-HSD1, we administered a selective 11β-HSD1 inhibitor in type 1 and type 2 murine models of diabetes and in cultured cardiomyocytes. Our results show that diabetes increases cortisone levels in heart tissues. 11β-HSD1 inhibitor decreased cortisone levels and ameliorated all structural and functional features of diabetic cardiomyopathy including fibrosis and hypertrophy. We also show that high levels of glucose caused cardiomyocyte hypertrophy and increased matrix protein deposition in culture. Importantly, inhibition of 11β-HSD1 attenuated these changes. Moreover, we show that 11β-HSD1 activation mediates these changes through modulating EGFR phosphorylation and activity. Our findings demonstrate that 11β-HSD1 contributes to the development of diabetic cardiomyopathy through activation of glucocorticoid and EGFR signaling pathway. These results suggest that inhibition of 11β-HSD1 might be a therapeutic strategy for diabetic cardiomyopathy, which is independent of its effects on glucose homeostasis.

## INTRODUCTION

Diabetes is well-established to increase the risk of heart failure. The pathological features of diabetic cardiomyopathy include cardiomyocyte hypertrophy and apoptosis, and interstitial fibrosis. The etiology of diabetic cardiomyopathy is largely due to hyperglycemia. Every 1% increase in HbA1c results in an 8% increased risk for developing heart failure [1]. Several biochemical aberrations contribute to the development of diabetic cardiomyopathy. These include reactive oxygen species, protein kinase C activation, production of advanced glycation end products, the renin-angiotensin-aldosterone system and mitochondrial dysfunction [2, 3]. However, effective treatments to prevent the development and/or progression of cardiomyopathy are still not available. Therefore, there is an urgent need to identify new therapeutic targets.

11β-HSD1 is a key enzyme that catalyzes the intracellular conversion of the inactive glucocorticoid cortisone to active cortisol (11-dehyrocorticosterone to corticosterone in rodents). Recent studies have suggested that 11β-hydroxysteroid dehydrogenase-1 (11β-HSD1) is an important risk factor for diabetes and diabetic complications [4–7]. Importantly, it is the activity of 11β-HSD1 that determines the intracellular concentration of active glucocorticoids in the tissues and not the plasma glucocorticoid levels [8]. Diabetes is associated with tissue-specific alterations in glucocorticoid metabolism, with increased activity of 11β-HSD1 reported in adipose tissue [9], liver [10], islets [11] and skeletal muscles [12]. Activation of 11β-HSD1 results in excess production of glucocorticoids and enhancement of glucocorticoid receptor (GR)-mediated insulin resistance and glucose intolerance. The importance of 11β-HSD1 in glucose homeostasis has been demonstrated in studies utilizing adipose tissue- and liver-specific 11β-HSD1 transgenic mice that show insulin resistance, hyperglycemia, hyperlipidemia, and hypertension [13–15]. In contrast, reduction in 11β-HSD1 activity by either 11β-HSD1 inhibitors or target gene disruption ameliorates features of metabolic syndrome. Studies have reported that deficiency in 11β-HSD1 results in lowering of body weight, and reduced levels of insulin, fasting glucose, triglyceride and cholesterol in type 2 diabetes mouse models [6]. Therefore, 11β-HSD1 has been recognized as a potential therapeutic target for type 2 diabetes and several 11β-HSD1 inhibitors have been evaluated in phase I or phase II clinic trials for the treatment of type 2 diabetes [16].

11β-HSD1 has recently been implicated in myocardial infarction, atherosclerosis, and angiogenesis [17, 18]. Myocardial infarction produced by coronary artery ligation in 11βHSD1(-/-) mice showed improved ejection fraction and higher microvessel density compared to wildtype mice [17]. Whether 11β-HSD1 plays a role in the pathogenesis of diabetic cardiomyopathy is, however, not known. Several studies have demonstrated that excessive glucocorticoids from glucocorticoid treatments or through increased activity of 11β-HSD1 are associated with increased risk for heart failure. Exogenous glucocorticoid therapy is associated with over a 2.5-fold increase of risk for heart failure [19]. In animal models of cardiac hypertrophy, 11β-HSD1 expression has been shown to double [20]. Moreover, administration of 11β-HSD1 inhibitor fully rescues myocardial hypertrophy [20]. Based on these interesting findings, we hypothesize that 11β-HSD1 mediates diabetes-induced cardiomyopathy. To test our hypothesis, we have used an animal model of diabetes as well as cultured cardiomyocytes. Our studies show that diabetes-induced cardiomyopathy is mediated through 11β-HSD1. Inhibition of 11β-HSD1 ameliorated structural and functional features of diabetic cardiomyopathy and that this beneficial effect was mediated through modulation of epidermal growth factor receptor phosphorylation.

## RESULTS

### 11β-HSD1 inhibitor prevents cardiac fibrosis and hypertrophy in mice with type 2 diabetes

**Figure 1:**
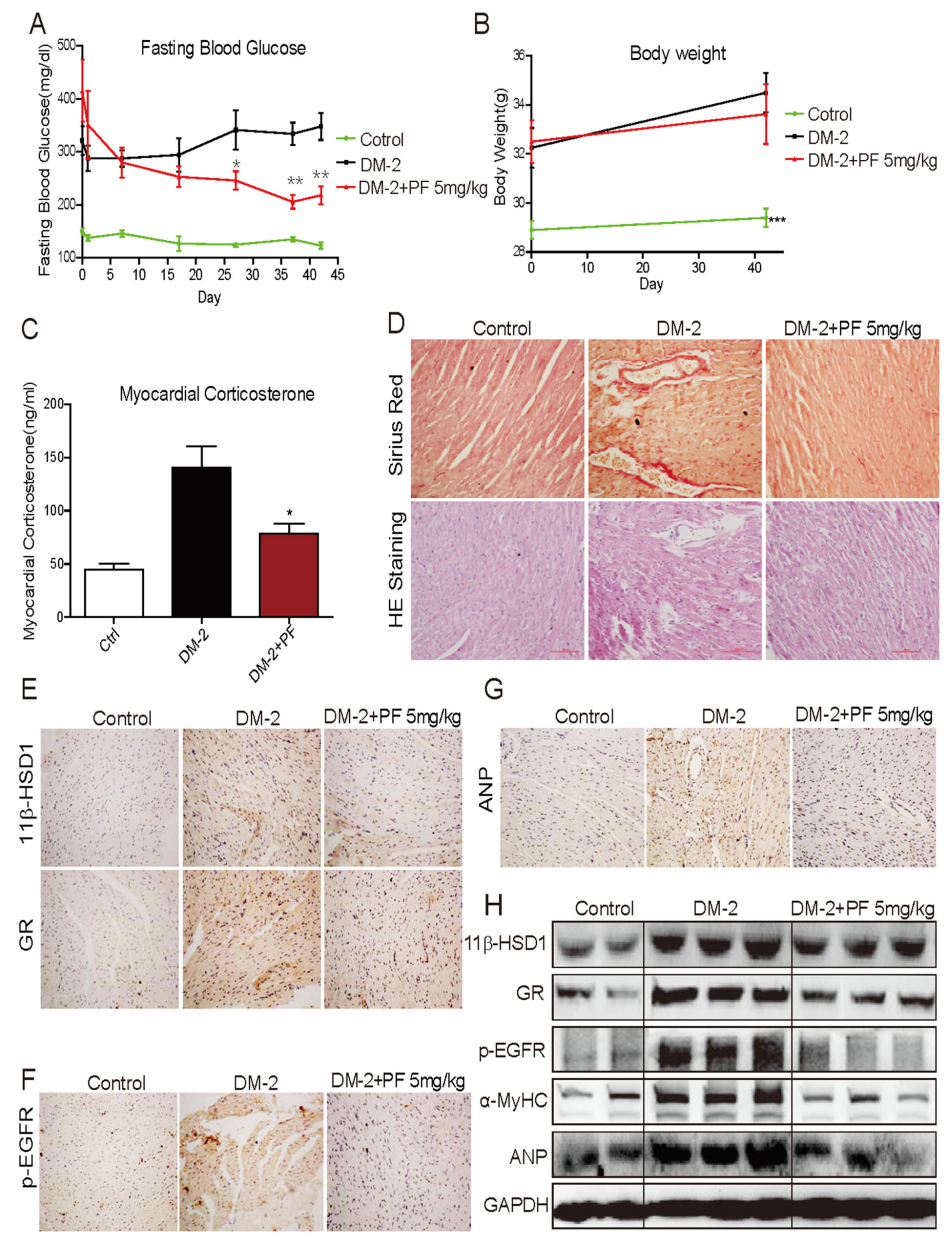
11β-HSD1 inhibitor PF-915275 lowered serum glucose levels and reduced pathological cardiac changes in a type 2 model of diabetes Diabetes was induced in male C57/BL6 mice by a single injection of low-dose STZ (30mg/kg body weight). Mice were then fed a high fat diet. Diabetic mice (fasting-blood glucose >12 mmol/L) were treated with PF-915275 (PF, 5 mg/kg; DM-2+PF) or vehicle (DM-2) by oral gavage once every 2 days for 6 weeks (n = 8 in each group). Control animals were injected with citrate buffer alone and fed standard chow (control). At the indicated time points, blood glucose **(A)** and body weight **(B)** were measured. **(C)** Corticosterone in myocardial tissue was determined using ELISA kit. **(D)** Representative images for the Sirius Red staining (upper panel) and Hematoxylin–Eosin (H&E, lower panel) staining of formalin-fixed myocardial tissues (400× original magnification). **(E-G)** Representative images for immunohistochemical staining of 11β-HSD1, GR, p-EGFR and ANP accumulation in formalin-fixed myocardial tissues as described in the methods section (brown color = DAB staining). **(H)** Western blot analysis for the expression of 11β-HSD1, GR, p-EGFR, α-MyHC and ANP protein in myocardial tissue of the different experimental groups. (n=8, *P<0.05 and ^**^P<0.01, versus DM group).

We first analyzed the type 2 diabetes model (DM-2). The fasting blood glucose levels in these diabetic mice significantly increased to more than 300 mg/dL (>16.6 mmol/L). Treatment of diabetic mice with PF-915275, a commercially available 11β-HSD1 inhibitor, significantly decreased blood glucose levels (Figure 1A). This was expected as studies have shown that inhibition of 11β-HSD1 improves glucose tolerance in diet-induced obese mice [21, 22]. However, we did not find any differences in body weights between diabetic mice and diabetic mice treated with PF (Figure 1B). We next measured corticosterone levels in heart tissues and observed significantly higher levels in diabetic mice compared to non-diabetic controls (Figure 1C). Administration of PF reduced the levels of corticosterone in mice as expected from inhibited 11β-HSD1. Sirius Red staining revealed increased collagen deposition in heart tissues of diabetic mice which was normalized by by PF treatment (Figure 1D). Irregular and disorganized muscle fibers in diabetic mouse hearts can be seen in H&E stained sections. Again, PF treatment normalized this alteration as the muscle fibers in DM-PF group were indistinguishable from non-diabetic controls (Figure 1D). Figure 1C suggests that diabetes induces 11β-HSD1. We confirmed this idea by assessing the expression of 11β-HSD1 and GR. The expression of both 11β-HSD1 and GR was significantly increased in diabetic mice compared to non-diabetic controls as measured by immunohistochemistry and western blotting (Figure 1E, 1H). PF treatment reduced the expression of both 11β-HSD1 and GR to a level comparable to non-diabetic mice. Furthermore, the expression pattern of ANP and α-MyHC (Figure 1G, 1H) followed the degree of fibrosis in heart tissues. Both ANP and α-MyHC are reliable markers of cellular hypertrophy and these findings show that the key features of diabetic cardiomyopathy (fibrosis and hypertrophy) are present in the low dose STZ-high fat model of type 2 diabetes and are prevented by 11β-HSD1 inhibition.

EGFR plays a key role in the development of cardiac hypertrophy [23–25] and it is also activated in diabetes [26–28]. It has been reported that glucocorticoids may increase the binding of EGF to EGFR [29]. Therefore, it is possible that diabetes-induced increased 11β-HSD1 and glucocorticoids mediate cardiac fibrosis through engaging the EGFR pathway. We tested the phosphorylation status of EGFR and show significantly increased levels in the heart tissues of diabetic mice by both immunostaining and western blotting (Figure 1F, 1H). We also show that treatment of diabetic mice with PF reduces the phosphorylation of EGFR.

### 11β-HSD1 inhibition ameliorates diabetes-induced cardiomyopathy independent of its glucose lowering effect

**Figure 2:**
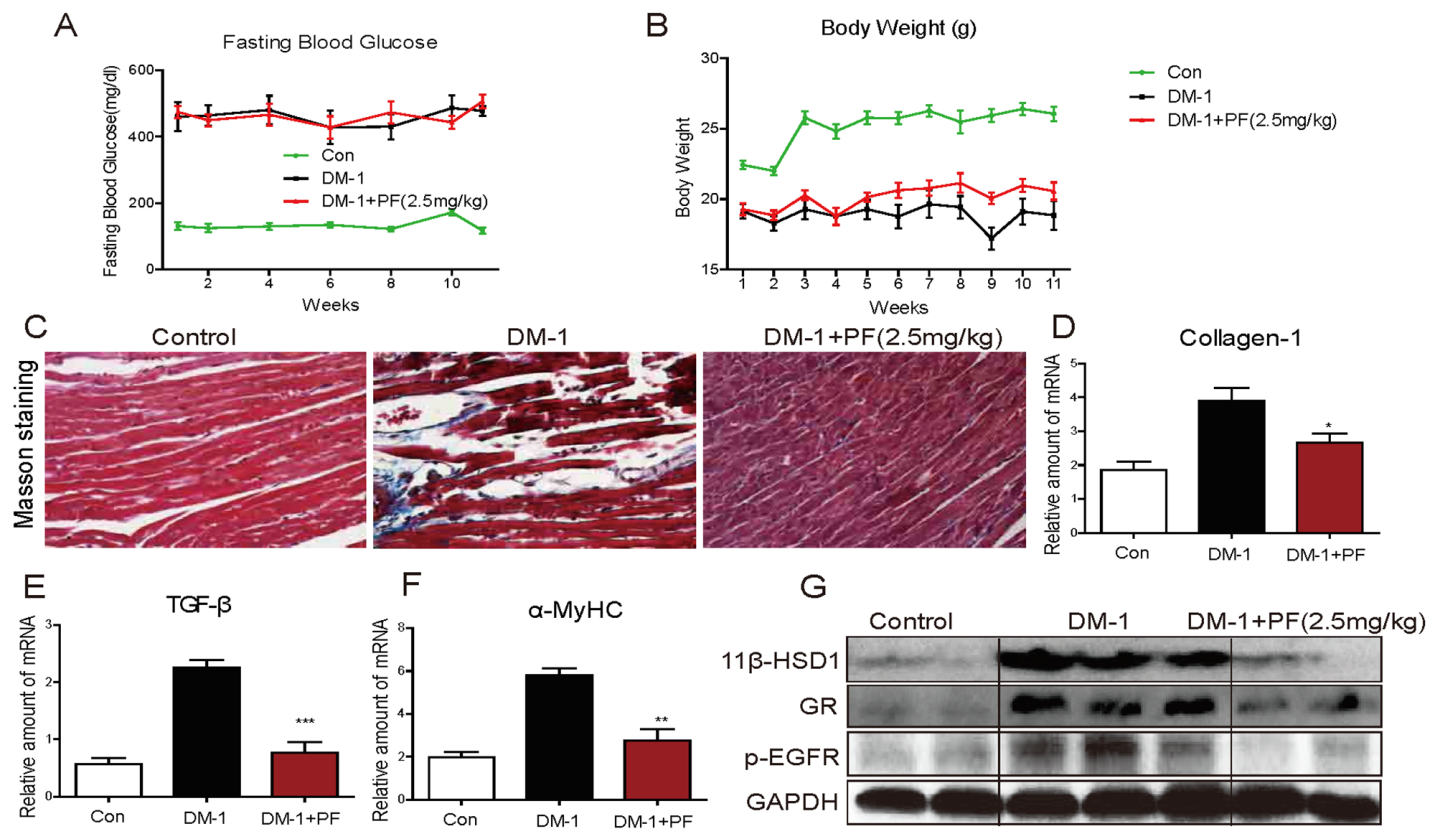
11β-HSD1 inhibition did not change serum glucose levels but reduced cardiac alterations in STZ-induced type 1 diabetic mice Diabetes was induced in male C57/BL6 mice by a single intraperitoneal (i.p.) injection of STZ (100mg/kg). Diabetic mice (blood glucose >12 mmol/L) were treated with 11β-HSD1 inhibitor PF-915275 (PF, 2.5 mg/kg) or vehicle by oral gavage once every 2 days for 11 weeks (n = 8 in each group). At the indicated time points, blood glucose **(A)** and body weights **(B)** were determined. **(C)** Representative images of Masson's trichrome stain are shown (blue color = collagen, black = nuclei, and red = cytoplasm). **(D-F)** mRNA levels of hypertrophic genes showing collagen1 (D), TGF-β (E), and α-MyHC (F). Six mice in each group were used for mRNA analysis. *P< 0.05, ^**^p < 0.01, ^***^p < 0.001, versus DM-1 group. **(G)** Western blot analysis showing expression of 11β-HSD1, GR and p-EGFR protein in myocardial tissue.

We showed that PF treatment prevented features of diabetic cardiomyopathy in a type 2 model of diabetes. PF treatment also lowered the blood glucose levels in these mice. 11β-HSD1 inhibition improves insulin sensitivity [8, 13, 14] and it is possible that the beneficial effects of PF are mediated, at least in part, through this hypoglycemic activity. To determine the extent to which beneficial effects of PF are attributable to insulin sensitivity, we utilized a type 1 diabetes model. In this model, a high dose STZ destroys pancreatic β cells removing possible confounding effects of PF on insulin sensitivity. Our results show that glucose levels in these mice are greater than 400 mg/dL (> 22 mmol/L) and PF treatment had no effect on glucose levels or body weights (Figure 2A, 2B). Collagen deposition was significantly increased in the heart of STZ-induced type 1 diabetic mice as demonstrated by Masson Trichrome staining (Figure 2C), Sirius Red staining (Supplementary Figure 1), and real-time qPCR (Figure 2D). Similar to our type 2 model, PF treatment reduced measures of cardiac fibrosis in type 1 diabetic mice. As a further measure of fibrosis, we analyzed the expression of transforming growth factor-β1 (TGF-β). The expression of TGF-β was significantly increased in the heart of STZ-induced diabetic mice and PF treatment blocked the increase (Figure 2E). Furthermore, expression of α-MyHC, 11β-HSD1, and GR followed the same pattern as in the type 2 model: increased expression in diabetic mice and reduced levels in the PF treated mice (Figure 2F, 2G). Lastly, we examined the level of p-EGFR in heart tissues and show significantly increased levels in the heart of diabetic mice (Figure 2G). PF treatment prevented diabetes-induced EGFR phosphorylation.

We examined cardiac function in the type 1 diabetes model to determine whether cardiac fibrosis and hypertrophy correlates with functional alterations. We performed non-invasive transthoracic echocardiograph one day before sacrifice. As shown in Table 1, diabetic mice showed a decrease in cardiac structure parameters (see IVS and LVPW; diastolic and systolic) and systolic function alterations (LVEF). These deficits were significantly attenuated in 11β-HSD1 inhibitor-treated mice. All cardiac structural and functional parameters in 11β-HSD1 inhibitor-treated diabetic mice were similar to non-diabetic control mice.

**Table 1:** Cardiac function in type 1 model of diabetes

Parameters	Control (N=8)	DM-1 (N=8)	DM-1+PF (N=8)
HR	541±13.2	510±32.0	554.3±20.1
IVS,d	0.86±0.05*	0.71±0.015	0.82±0.009*
LVID,d	2.55±0.05	2.4±0.059	2.5±0.07
LVPW,d	0.84±0.028*	0.65±0.013	0.81±0.01*
IVS,s	1.20 ±0.076*	0.84±0.017	0.99±0.008*
LVID,s	1.43±0.033*	1.87 ±0.016	1.58 ±0.12*
LVPW,s	1.25±0.04*	0.95±0.016	1.12±0.008
Fractional shortening (%)	47.65±3.928*	37.3±2.78	43.6±4.47*
LVEF (%)	81.03±1.92*	69.2±5.15	79.1±3.51*

Data shown as Mean ± SEM, n = 8 per group; *p<0.05 vs. DM-1 group. HR: heart rate; IVS: the interventricular septum, diastole; LVID: the left ventricular interior diameter; LVPW: the left ventricle posterior wall; d: diastole; s: systole. Diastolic function was assessed using pulsed-wave Doppler imaging of the transmitral filling pattern. Ejection fraction (LVEF) was calculated from LV end-diastolic volume (LVEDV) and end-systolic volume (LVESD) using the equation of (LVEDV-LVESV)/LVEDV × 100%. Fractional shortening (FS) was calculated using the equation (FS = [(LVIDd -LVIDs)/LVIDd] × 100).

### Inhibition of 11β-HSD1 blocks high glucose-induced cardiomyocyte hypertrophy and EGFR phosphorylation

**Figure 3:**
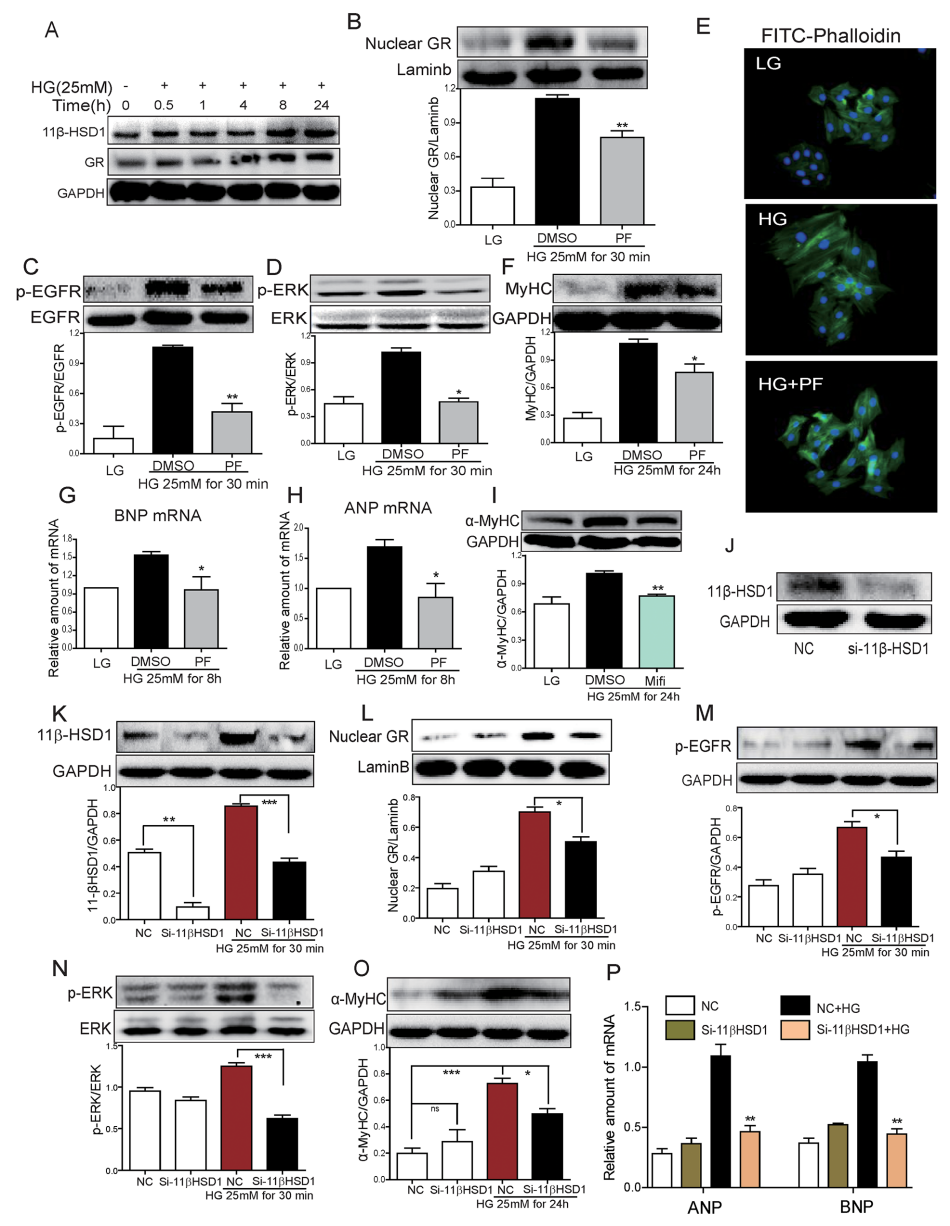
Inhibition of 11β-HSD1 by PF or small interfering RNA attenuated HG-induced 11β-HSD1/GR, EGFR phosphorylation and cardiomyocyte hypertrophy **(A)** H9c2 cardiomyocytes was stimulated with HG (25mM) for different time periods. Western blot analysis was then used to examine the expression of 11β-HSD1 and GR. **(B-D)** H9c2 cells were pretreated with DMSO or PF-915275 (10 μM) for 2 h followed by incubated with HG (25mM) for 30 mins. Western blot analysis was then used to determine levels of nuclear GR (B), EGFR phosphorylation (C) and ERK phosphorylation (D). Bar graphs below the blots show densitometry quantification from three independent experiments (*P < 0.05, ^**^P < 0.01, versus HG group). **(E)** H9c2 cells were pretreated with DMSO or PF (10 μM) for 2 h, then stimulated with HG (25mM) for 24 h. Representative images for FITC-phalloaidin staining are shown. **(F)** H9c2 cells were pretreated with DMSO or PF (10 μM) for 2 h, then stimulated with HG (25mM) for 24 h. α-MyHC was detected by western blotting. Upper panel showed representative western blot images and lower panel showed the densitometry quantification (*P < 0.05, versus HG group). **(G** and **H)** H9c2 cells were pretreated with DMSO or PF (10 μM) for 2 h followed by incubated with HG (25mM) for 8 hour. mRNA level of BNP and ANP were determined by qPCR and normalized to β-actin. Each bar represents mean ± SD of three to five independent experiments (* P <0.05, versus HG group). **(I)** H9c2 cells were pretreated with Mifepristone (Mifi, 10 μM) for 2 h, followed by stimulating with HG (25mM) for 24 h. α-MyHC was detected by western blotting (^**^P < 0.01, versus HG group). (J-P) H9C2 cells were transfected with 11β-HSD1 siRNA **(J)** and then stimulated with HG (25mM) for 0.5 h, 8 h, or 24 h (depending on the mRNA/protein for analysis). Figures showing 11β-HSD1 protein at 0.5 h **(K)**, nuclear GR protein at 0.5 h **(L)**, phospho-EGFR at 0.5 h **(M)**, phospho-ERK at 0.5 h **(N)**, α-MyHC protein at 24 h **(O)**, and ANP and BNP mRNA at 8 h **(P)**. Each bar represents mean ± SD of three independent experiments (*p< 0.05, ^**^p < 0.01, ^***^p < 0.001, versus si-11β-HSD1 HG group).

Our next objective was to investigate the mechanisms by which PF prevents diabetic cardiomyopathy. To delineate these mechanisms, we used cultured H9c2 cardiomyocyte line exposed to high levels of glucose to mimic diabetes. Our data shows that high glucose (HG; 25 mM) increased 11β-HSD1 and GR protein expression in cardiomyocytes in a time-dependent manner (Figure 3A). Increased expression was evident as early as 30 minutes of HG exposure and these levels were maintained for 24 hours. In addition to increased expression, GR showed increased nuclear localization at 30 mins (Figure 3B) and this increase was prevented with 2-hour PF pre-treatment. These results indicate that HG increases 11β-HSD1 expression, which leads to GR nuclear localization. As our *in vivo* studies pointed to an interesting involvement of EGFR in diabetic cardiomyopathy, we investigated whether HG increases EGFR phosphorylation. Indeed, HG increased the phosphorylation of EGFR and its downstream signaling protein extracellular signal-regulated kinase (ERK) (Figure 3C, 3D). Importantly, the effect of HG on EGFR and ERK phosphorylation was blunted by PF pretreatment, indicating that 11β-HSD1 mediated the effects of HG on EGFR and ERK phosphorylation. In addition to EGFR phosphorylation, stimulation with HG caused cellular hypertrophy (Figure 3E) and increased the expression of hypertrophic markers α-MyHC, ANP and BNP (Figure 3F-3H). These changes were also prevented by inhibiting 11β-HSD1.

11β-HSD1 catalyzes the conversion of inactive glucocorticoid cortisone to active cortisol (corticosterone) which binds with GR and causes its nuclear translocation. We determined whether blocking GR activity would prevent HG-induced cellular hypertrophy. We used a competitive inhibitor of GR, Mifepristone (Mifi), which antagonizes active glucocorticoid action competitively at the receptor level. Our results show that the HG-induced upregulation of α-MyHC was inhibited by preventing GR signaling through Mifi (Figure 3I). We confirmed this pathway by knocking down the expression of 11β-HSD1 in cardiomyocytes by siRNA. siRNA transfection led to a 50% reduction in the expression of 11β-HSD1 in H9c2 cells (Figure 3J). Consistent with the effects of PF treatment, knockdown of 11β-HSD1 blocked the effects of HG on GR nuclear translocation, EGFR and ERK phosphorylation and the expression of α-MyHC, ANP and BNP (Figure 3K-3P).

### Cortisone (CORT) causes cardiomyocyte hypertrophy through 11β-HSD1

**Figure 4:**
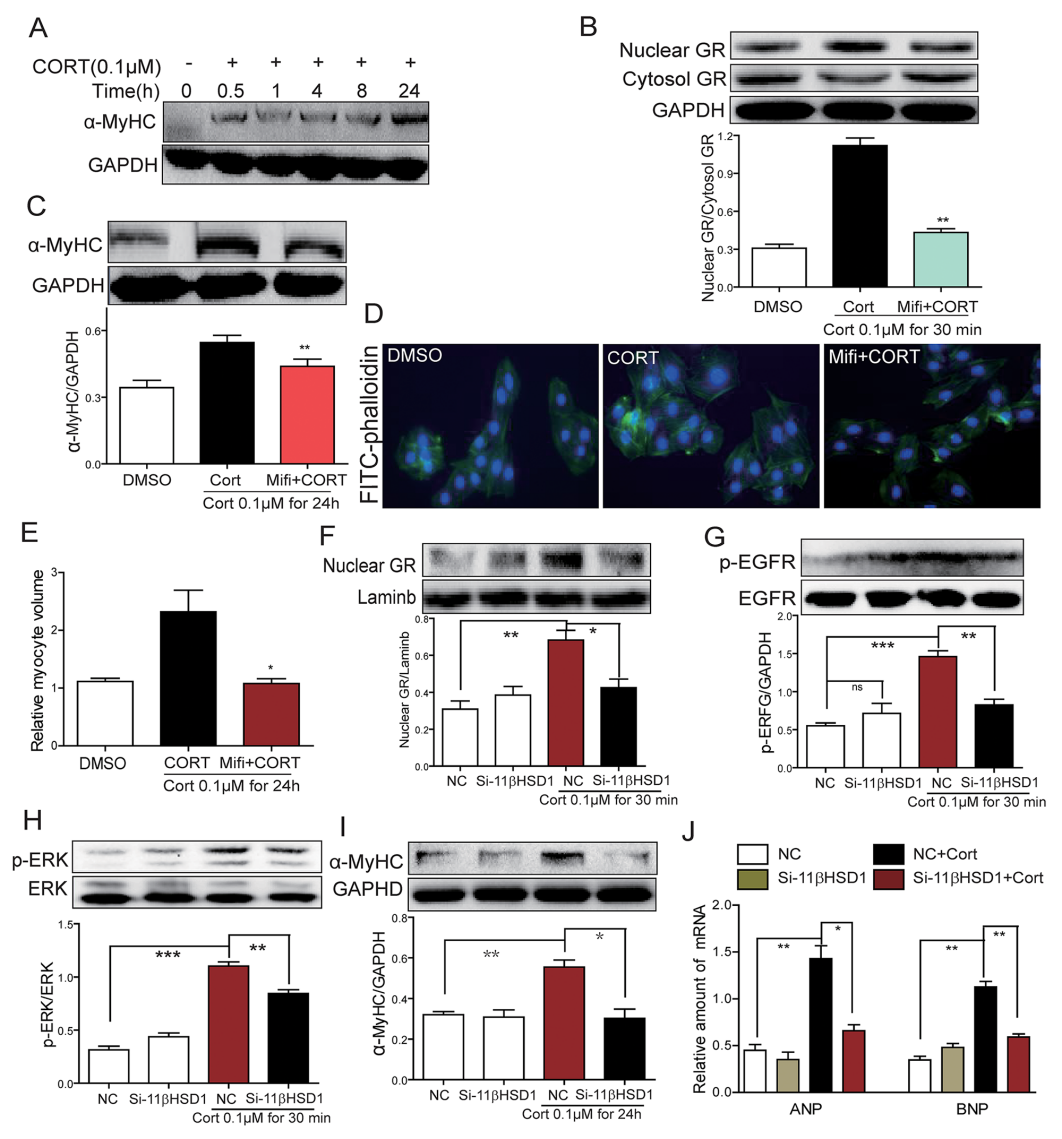
Inhibition of GR or inhibition of 11β-HSD1 prevents cortisone-induced 11β-HSD1, GR translocation, EGFR phosphorylation and cardiomyocyte hypertrophy **(A)** H9c2 cells were stimulated with cortisone (0.1μM; CORT) for different various time periods. Western blot analysis was then used to examine α-MyHC. **(B-C)** H9c2 cells were pretreated with Mifepristone (10 μM; Mifi) for 2 h and stimulated with cortisone (0.1 μM) for 30 min or 24h. Western blot analysis was then used to examine both cytosolic and nuclear GR (B, at 30 min), and α-MyHC (C, at 24h). Upper panel showed representative Western blotting images and lower panel showed the quantification (^**^P < 0.01, versus CORT group). (D-E) H9c2 cells were pretreated with Mifi (10 μM) for 2 h, then stimulated with cortisone (0.1 μM) for 24 h. **(D)** Representative images for FITC-phalloidin staining are shown. **(E)** Quantification of cell volume. Each bar represents the mean ± SD of three independent experiments (*P < 0.05, versus CORT group). **(F-J)** H9C2 cells were transfected with 11β-HSD1 siRNA and stimulated with cortisone (0.1 μM) for 0.5 h, 8 h, or 24 h. Figure showing nuclear GR protein (F, at 0.5 h), phospho-EGFR protein (G, at 0.5 h), phospho-ERK protein (H, at 0.5 h), α-MyHC protein (I, at 24 h), and mRNA levels of ANP and BNP mRNA (J, at 8 h). Each bar represents mean ± SD of three independent experiments (*p< 0.05, ^**^p < 0.01, ^***^p < 0.001, versus si-11β-HSD1 CORT group).

We have shown that 11β-HSD1 is necessary for HG-induced cardiomyocyte hypertrophy. We reasoned that cortisone would mimic the effects of HG by activating 11β-HSD1 and GR signaling. We treated H9c2 cells with cortisone (CORT) and show induction of α-MyHC expression in a time-dependent manner (Figure 4A). Increased α-MyHC expression was seen at 30 minutes after CORT stimulation and reached peak levels at 24 hours after stimulation. CORT stimulation also increased nuclear GR localization which was blocked by Mifi (Figure 4B). In addition, CORT induced an upregulation of α-MyHC (Figure 4C) and caused cardiomyocytes hypertrophy (Figure 4D). Pretreatment with Mifi abolished the effects of CORT on α-MyHC expression and cardiomyocyte hypertrophy (Figure 4D and 4E). These effects of CORT are believed to be mediated through 11β-HSD1 activity and production of active cortisol. If true, knocking down the expression of 11β-HSD1 would mitigate the effects of CORT. Indeed, our data shows that 11β-HSD1 silencing prevents CORT-induced GR nuclear translocation, phosphorylation of EGFR and ERK, and increased expression of α-MyHC, ANP and BNP (Figure 4E-4J). These results clearly demonstrate that HG induced 11β-HSD1 expression, increased active glucocorticoid levels, and GR translocation to mediate cellular hypertrophy. The results further implicate EGFR in this pathway.

### GR is required for high glucose-induced EGFR phosphorylation and cardiomyocyte hypertrophy

**Figure 5:**
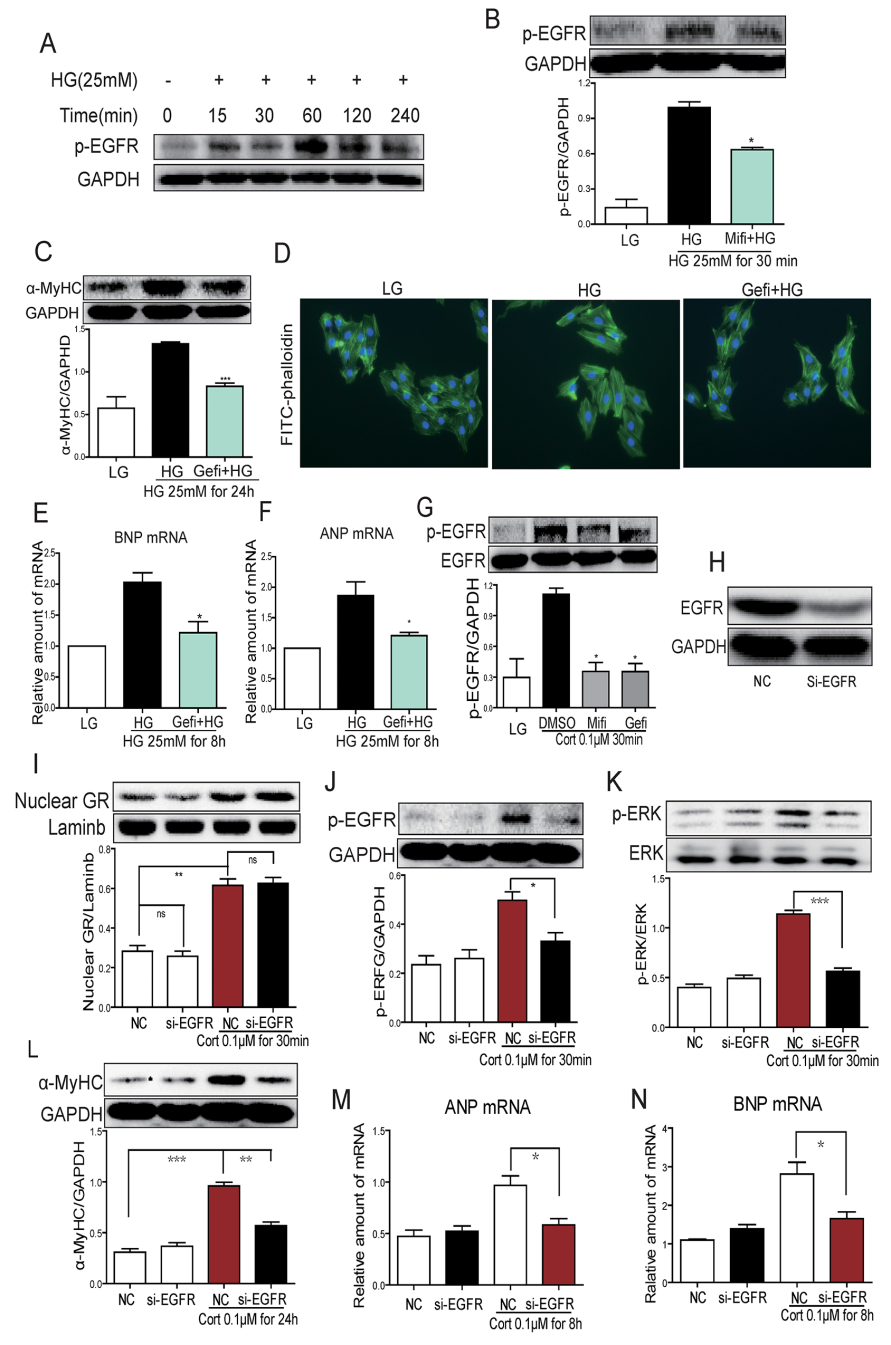
HG induces EGFR activation through 11β-HSD1-GR signaling in H9c2 cells **(A)** H9c2 cells were stimulated with HG (25 mM) for different time periods and western blot analysis was used to examine p-EGFR. **(B)** H9c2 cells were pretreated with Mifi (10 μM) for 2 h and stimulated with HG (25 mM) for 30 min. Western blot analysis was used to examine p-EGFR. Upper panel show representative blot images and lower panel show the quantification (*P < 0.05, versus HG group). **(C-F)** H9c2 cells were pretreated with Gefitinib (Gefi, 10 μM) for 2 h and stimulated with HG (25 mM) for 30 min, 8h or 24h. Assessment of cell hypertrophy showing α-MyHC protein (C, at 24 h), representative FITC-phalloidin staining (D), BNP mRNA (E, at 8h) and ANP mRNA (F, at 8 h). Each bar represents mean ± SD of three independent experiments (*p< 0.05, ^**^p < 0.01, ^***^p < 0.001, versus si-11β-HSD1 HG group). **(G)** H9c2 cells were pretreated with Gefitinib (Gefi, 10 μM) or Mifepristone (Mifi, 10 μM) for 2 h and stimulated with cortisone (0.1 μM) for 30 min. p-EGFR protein was determined by western blot. Data is shown as mean ± SD of three independent experiments (*p< 0.05, versus CORT group). **(H-N)** H9C2 cells were transfected with EGFR siRNA and then stimulated with cortisone (0.1μM) for 0.5 h, 8 h, or 24 h. Figures showing EGFR protein (H, at 0.5 h), nuclear GR protein (I, at 0.5 h), p-EGFR protein (J, at 0.5 h), p-ERK protein (K, at 0.5 h), α-MyHC protein (L, at 24 h), and mRNA levels of ANP (M, at 8 h) and BNP (N, at 8 h). Each bar represents mean ± SD of three independent experiments (*p< 0.05, ^**^p < 0.01, ^***^p < 0.001, versus si-EGFR CORT group).

Our last objective was to place EGFR in HG-induced GR signaling and cellular hypertrophy process. We tested whether GR was dispensable for EGFR phosphorylation induced by HG or CORT. Cells were stimulated with HG with or without the presence of CORT and EGFR alteration was assessed. As shown in Figure 5A, HG increased phospho-EGFR levels in a time-dependent manner. Pretreatment with Mifi reduced HG-induced EGFR phosphorylation (Figure 5B). To directly link HG-induced EGFR activity to cardiomyocyte hypertrophy, we used a potent and selective EGFR inhibitor Gefitinib (Gefi). Pretreatment with Gefi significantly inhibited HG-induced α-MyHC, ANP and BNP upregulation as well as cardiomyocyte hypertrophy (Figure 5C-5F). Next, we linked EGFR phosphorylation to GR signaling and show that CORT-induced EGFR phosphorylation could be blocked by Mifi and Gefi (Figure 5G). However, knockdown of EGFR by siRNA (Figure 5H) could not prevent CORT-induced GR translocation but reduced EGFR and ERK phosphorylation, as well as α-MyHC, ANP and BNP upregulation (Figure 5J-5N). These results indicate that EGFR may be downstream of GR translocation in CORT-induced cardiomyocyte hypertrophy.

## DISCUSSION

In this study, we have demonstrated that 11β-HSD1 and GR mediate diabetes-induced cardiomyopathy. We show that high glucose levels activate 11β-HSD1, which leads to GR nuclear translocation, and EGFR activity resulting in cardiomyocyte hypertrophy.

We studied diabetic cardiomyopathy in two diabetic animal models. First, type 2 diabetes was induced by combining low-dose STZ and high fat feeding [30, 31]. This model shows impaired glucose tolerance, markedly reduced insulin levels, and elevated HbA1c levels. In our study, we show hallmark features of diabetic cardiomyopathy in these mice evidenced by increased myocardial fibrosis and increased expression of cardiomyocyte hypertrophy markers. Heart tissues also showed increased expression of 11β-HSD1 and GR, as well as increased cardiac glucocorticoid levels, compared to non-diabetic mice. Administration of 11β-HSD1 inhibitor decreased fibrosis and the expression of ANP in the heart, indicating that 11β-HSD1 inhibitor rescues diabetic cardiomyopathy. Although these results are convincing, one caveat is that 11β-HSD1 inhibition improves insulin sensitivity which may mask the direct beneficial effects of 11β-HSD1 inhibition of cardiomyopathy. To circumvent this, we utilized a type 1 model which lacks insulin production. In this model, 11β-HSD1 inhibitor also ameliorated myocardial fibrosis and expression of hypertrophic markers. Together, our *in vivo* studies strongly suggest that diabetes causes cardiac dysfunction through 11β-HSD1 and this can be prevented through 11β-HSD1 inhibition. Results of the type 1 diabetes model also show that the beneficial effects of inhibiting 11β-HSD1 are independent of its effects on glucose homeostasis.

To dissect the role of 11β-HSD1/GR axis in diabetic cardiomyopathy, we performed *in vitro* studies in the H9c2 cardiomyocyte line. Glucose levels similar to diabetic conditions resulted in increased cardiomyocyte cell size and upregulation of ANP and BNP, two classic markers for cardiomyocyte hypertrophy. In this model system, the important role of 11β-HSD1/GR axis was supported by several lines of evidence. First, expression of 11β-HSD1and GR was upregulated by high glucose in cultured cardiomyocytes. A similar phenomenon is also reported in hepatocytes in which high glucose increases 11β-HSD1 and GR expression [10]. Second, inhibition of 11β-HSD1 blocked high glucose-induced increase in cell size and upregulation of ANP and BNP. Third, CORT (inactive glucocorticoid) treatment could also increase the expression of ANP and BNP in cardiomyocytes. And these changes are prevented by inhibiting 11β-HSD1. And finally, knockdown of 11β-HSD1 or blocking of GR rescues high glucose-induced cardiomyocyte hypertrophy. These studies clearly show that 11β-HSD1 promotes the development of diabetic cardiomyopathy. We further showed that high glucose and CORT promote GR nuclear translocation. Inhibition of GR translocation blocks high glucose-induced increases in ANP and BNP, indicating the nuclear translocation of GR is downstream of 11β-HSD1 activation.

An interesting finding of the *in vivo* studies was the association of EGFR phosphorylation with diabetes-induced 11β-HSD1 pathway and downstream cardiac dysfunction. EGFR is a receptor tyrosine kinase and EGFR activation has been shown to play an important role in the development of cardiac hypertrophy [32, 33]. We previously reported that EGFR inhibition attenuates diabetic cardiomyopathy in mice [34]. G-protein coupled receptor (GPCR) agonists, such as phenylephrine, angiotensin II and endothelin-1 are well-known inducers of cardiac hypertrophy. These GPCR agonists activate disintegrin and metalloprotease 12 (ADAM12, a membrane-bound enzyme), which causes the shedding of heparin-binding epidermal growth factor (HB-EGF). HB-EGF is then able to trans-activate EGFR and promote cardiac hypertrophy. Inhibition of HB-EGF shedding attenuates cardiac hypertrophy induced by GPCR agonists indicating a key role of EGFR activation in the process [35]. High levels of glucose have also been reported to increases EGFR N-glycosylation and activity in vascular smooth muscle cells [36]. In agreement with previous reports in other organs, we also found that EGFR phosphorylation is increased in the heart of diabetic mice as well as in cultured cardiomyocytes exposed to high levels of glucose. Interestingly, 11β-HSD1 inhibition can prevent EGFR phosphorylation in both systems. Furthermore, stimulation of cardiomyocytes with corticosterone also increased EGFR phosphorylation. Again, inhibition of 11β-HSD1 or blocking GR prevents EGFR phosphorylation. A critical involvement of EGFR was demonstrated when we inhibited EGFR through a chemical inhibitor or knocked down the expression using siRNA and observed that EGFR deficit blocked high glucose- and CORT-induced cardiomyocyte hypertrophy. These data strongly suggest that 11β-HSD1 activation and subsequent GR nuclear translocation were indispensable for high glucose or CORT induced EGFR phosphorylation and subsequent development of diabetic cardiomyopathy.

One limitation of the present study is that we have not delineated how 11β-HSD1/GR increases EGFR phosphorylation in cardiomyocytes. A previous study reported that the binding of EGF to EGFR was increased after dexamethasone treatment, which might due to dexamethasone-mediated change in the micro-domain of cell surface [37]. It is also possible that dexamethasone increases EGFR expression thereby providing more binding sites for EGF. Another exciting possibility is that GR may induce ADAM12. Studies in chondrocytes show increased expression of ADAM12 upon dexamethasone treatment [38]. ADAM12 is also induced during adipocyte differentiation [39]. A critical component of this adipocyte differentiation inducing media formulation is dexamethasone. Therefore, it is possible that diabetes and high levels of glucose increase local glucocorticoid levels which induce ADAM12 and increase EGF-EGFR interaction leading to EGFR phosphorylation and activity. These interesting avenues should be the point of focus of future studies.

In summary, we found that inhibition of 11β-HSD1 preserves cardiac function and normalizes structural hallmarks of diabetic cardiomyopathy in type 1 and type 2 models. We also found that this prevention is mediated through modulation of EGFR phosphorylation. Thus, 11β-HSD1 could have beneficial effect in protecting the development of diabetic cardiomyopathy besides its hypoglycemic effects.

## MATERIALS AND METHODS

### Reagents and cell culture

Glucose and cortisone were purchased from Sigma-Aldrich (Louis, MO). Selective inhibitor of 11β-HSD1 PF-915275 [22] was purchased from Santa Cruz Biotechnology (Santa Cruz, CA). Epidermal growth factor receptor inhibitor Gefitinib [40–42] and GR antagonist Mifepristone [43, 44] were obtained from Sigma. PF-915275, Gefitinib and Mifepristone were dissolved in DMSO for *in vitro* studies and in CMC-Na (1%) for *in vivo* experiments. Antibodies for 11βHSD1, GR, α-myosin heavy chain (α-MyHC) and atrial natriuretic peptide (ANP) were purchased from Santa Cruz Biotechnology. Antibodies for extracellular signal-regulated kinase (p-ERK, ERK) and epidermal growth factor receptor (p-EGFR, EGFR) were obtained from Cell Signaling (Danvers, MA). H9c2 embryonic rat heart-derived cardiomyocyte line was obtained from the Shanghai Institute of Biochemistry and Cell Biology (Shanghai, China). Cells were cultured in DMEM medium containing 5.5 mmol/L of D-glucose supplemented with 10% FBS, 100 U/mL of penicillin, and 100 mg/mL of streptomycin. For high glucose treatments, cells were cultured in DMEM medium containing 25 mmol/L of glucose (high glucose, HG). In the cortisone-treated group, cells were treated with 0.1 μM of cortisone in DMEM media.

### Animal studies

Mice were obtained from Animal Center of Wenzhou Medical University. Protocols used for all animal studies were approved by the Wenzhou Medical University Animal Policy and Welfare Committee.

Type 1 diabetes model was produced by administering male C57BL/6 mice (8-12 weeks; weighing 23-25 g) a single intraperitoneal injection of streptozotocin (STZ, Sigma Chemicals) at the dose of 100 mg/kg. STZ was dissolved in 100 mM citrate buffer (pH 4.5) which was also used as vehicle control in non-diabetic mice. One week later, blood glucose levels were measured using a glucometer by tail vein puncture sampling. Mice with fasting-blood glucose levels greater than 12 mmol/L were considered diabetic and used in this study. 16 STZ mice were randomly divided into two groups and then started to be treated with PF or vehicle (Day 0)

To produce a type 2 diabetes model, we utilized low-dose STZ with high fat diet feeding model [30, 31]. Briefly, 16 male C57BL/6 mice were fed with a high fat diet (HFD) for 2 mice while 8 control mice were fed with standard chow. Then, we injected STZ at a dose of 30 mg/kg in HFD-fed mice. One week later, HFD-fed mice with fasting-blood glucose levels greater than 12 mmol/L were considered diabetic and used in this study. The diet contained 60 kcal.% fat, 20 kcal.% protein and 20 kcal.% carbohydrate (Cat.#MD12033; MediScience Diets Co. LTD, Yangzhou, China). Non-diabetic controls were fed the standard chow containing 10 kcal.% fat, 20 kcal.% protein and 70 kcal.% carbohydrate (Cat. #MD12031). 16 HFD-fed mice were randomly divided into two groups and then started to be treated with PF or vehicle (Day 0).

All mice had free access to food and water at all times. STZ- DM1 (type 1 model) received 11β-HSD1 inhibitor PF-915275 (2.5 mg/kg) or vehicle (CMC-Na) by oral gavage once every two days for 11 weeks (n=8 per group). STZ- DM2 (type 2) were treated with PF-915275 (5 mg/kg) or vehicle (CMC-Na) for 6 weeks (n=8 per group). At the indicated time points, blood glucose was determined and body weights recorded. Mice were anesthetized with 100 mg/kg ketamine hydrochloride (Ketanest, Pfizer, Germany) and 16 mg/kg xylazine hydrochloride (Rompun 2%, Bayer, Germany). Mice were sacrificed under anesthesia. Body weight was recorded and blood samples were collected and centrifuged to prepare serum. Heart tissues were embedded in 4% paraformaldehyde for histological analysis and/or snap-frozen in liquid nitrogen for gene and protein expression analyses. Corticosterone levels in heart tissues were measured by commercial ELISA kit from Nanjing Jiancheng Bioengineering Institute (Jiangsu, China).

### Cardiac function

Cardiac function was determined non-invasively by transthoracic echocardiography one day before sacrifice [45, 46]. Mice was anesthetized using isoflurane and echocardiography was performed by SONOS 5500 ultrasound (Philips Electronics, Amsterdam, Netherland) with a 15-MHz linear array ultrasound transducer.

### Cell transfections

EGFR and 11β-HSD1 expression was knocked down using siRNA. EGFR (5’-CCGUGCCUGAAUAUA UAAATT-3’ and 5’-UUUAUAUAUUCAGGCACGGTT-3’) and 11β-HSD1 (5’-GAGGUAUACUAUGACAAAUTT-3’ and 5’- AUUUGUCAUAGUAUACCUCTT-3’) siRNAs were purchased from Gene Pharama (Shanghai, China). Cells were transfected using LipofectAMINE 2000 (Thermo Fisher).

### Western blot analysis

Total proteins from heart tissues and total proteins as well as nuclear extracts from cultured cells were prepared. Nuclear proteins were prepared using Nuclear Translocation Assay Kit from Beyotime Biotechnology (China) as shown previously [45]. The protocols for routine western blot experiments are described in previous publications [35]. The density of the immunoreactive bands was analyzed using Image J software (NIH, Bethesda, MD, USA).

### Real-time quantitative PCR

Total RNA was isolated from heart tissues using TRIZOL (Thermo Fisher). Reverse transcription and quantitative PCR (RT-qPCR) were performed using M-MLV Platinum RT-qPCR Kit (Thermo Fisher) in Eppendorf Realplex4 (Eppendorf). Primers for genes were obtained from Thermo Fisher. Sequences for primers are provided in Supplementary Table 1. The relative amount of target mRNA was normalized to the amount of β-actin.

### Histology

Heart tissues were embedded in paraffin. Five μm thick sections were prepared and placed on positively charged slides. For histological analysis, tissue sections were deparaffinzed, rehydrated, and subjected to routine H&E staining. Sections were also stained with Masson's trichrome to highlight connective tissue and Sirius Red to stain collagen as shown by us previously [47]. For immunohistochemistry, paraffin sections were deparaffinized/rehydrated and subjected to antigen retrieval in 0.01 mol/L citrate buffer (pH 6.0) for 3 min at 98°C. Slides were then placed in 3% hydrogen peroxide in methanol for 30 min. After blocking with 5% bovine serum albumin (BSA) for 30 min, slides were incubated with primary antibodies against 11β-HSD1, GR, α-MyHC and ANP at 1:200 dilutions overnight at 4 °C. The next day, appropriate secondary antibodies (1:200, Santa Cruz) were used with DAB to visualize immunoreactivity. Slides were counterstained with hematoxylin. Images were taken using a Nikon microscope (Nikon, Japan).

### Cytoskeleton F-actin staining

H9c2 cells were fixed in 4% formalin and incubated with FITC-labeled phalloidin for 30 min. DAPI (4’,6-diamidino-2-phenylindole) was used to visualize nuclei. Cells were imaged using a Nikon epi-fluorescence microscope equipped with a digital camera (Nikon, Japan). Cell size was measured by image J and shown as arbitrary pixel units.

### Statistical analysis

The studies were randomized and blinded, and data reported as mean±SEM from at least three independent experiments. Statistical analysis was performed with GraphPad Prism 5.0 software (GraphPad, San Diego, CA, USA), using one-way ANOVA followed by Dunnett's post-hoc test when comparing more than two groups of data. When comparing two groups, the unpaired t-test was used. Statistical significance is defined at P value < 0.05.

## SUPPLEMENTARY MATERIALS FIGURES AND TABLES


