# Supplementary material for: 11β-HSD1 inhibition ameliorates diabetes-induced cardiomyocyte hypertrophy and cardiac fibrosis through modulation of EGFR activity
Source: Oncotarget. 2017 Oct 24;8(56):96263–75. doi: 10.18632/oncotarget.22015 (PMC5707098; doi:10.18632/oncotarget.22015)
Supplement: Supplementary file 1 [file oncotarget-08-96263-s001.pdf]

11β-HSD1 inhibition ameliorates diabetes-induced cardiomyocyte hypertrophy and cardiac fibrosis through modulation of EGFR activity

SUPPLEMENTARY MATERIALS

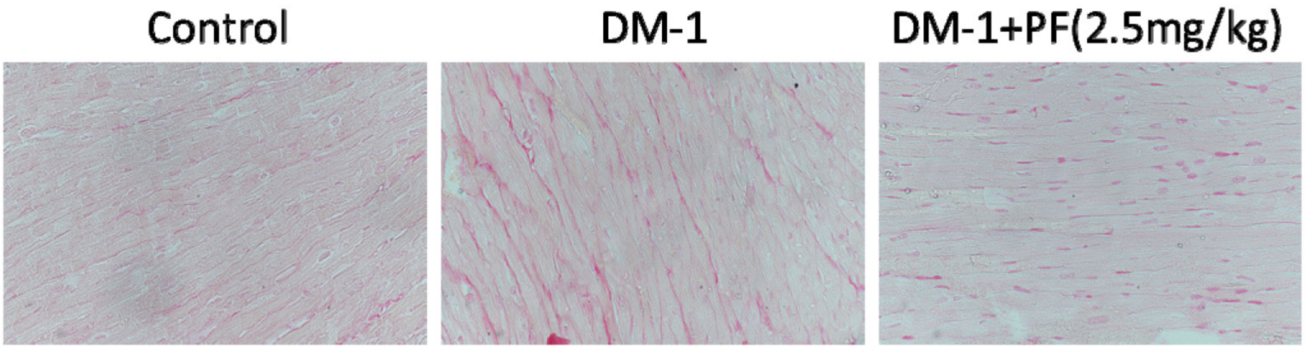

Supplementary Figure 1: PF reduced cardiac fibrosis in STZ-induced type 1 diabetic mice. Diabetes was induced in male C57/BL6 mice by a single intraperitoneal (i.p.) injection of STZ (100mg/kg). Diabetic mice (blood glucose >12 mmol/L) were treated with 11β-HSD1 inhibitor PF-915275 (PF, 2.5 mg/kg) or vehicle by oral gavage once every 2 days for 11 weeks (n = 8 in each group). Representative images of Sirius Red staining for heart tissue sections are shown (red color = collagen).

Supplementary Table 1: Primers used in real-time qPCR assay

Gene	Species	FW	RW
ANP	Rat	GAGGAGAAGATGCCGGTAG	TCAGAGAGGGAGCTAAGTG
BNP	Rat	TTCCGGATCCAGGAGAGACTT	CCTAAAACAACCTCAGCCCGT
β-Actin	Rat	AAGTCCCTCACCCTCCCAAAAG	AAGCAATGCTGTCACCTTCCC
TGF-β	Mouse	TGACGTCACTGGAGTTGTACGG	GGTTCATGTCATGGATGGTGC
Collagen1	Mouse	TGGCCTTGGAGGAAACTTTG	CTTGGAAACCTTGTGGACCAG
α-MyHC	Mouse	CGAGTCCCAGGTCAACAAG	AGGCTCTTTCTGCTGGACA
β-Actin	Mouse	CCGTGAAAAGATGACCCAGA	TACGACCAGAGGCATACAG
